# The association of dietary methionine, natural folate and folic acid supplementation with the risk of preterm birth during pregnancy: a case control study

**DOI:** 10.1186/s12889-026-28702-5

**Published:** 2026-08-07

**Authors:** Xinyue Zhou, Wenhui Wu, Zifu Wang, Lei Cao, Yuqing Li, Baohong Mao, Xuhong Chang

**Affiliations:** 1 School of Public Health, Lanzhou University, Lanzhou, 730000 China; 2 School of Public Health, Gansu University of Chinese Medicine, Gansu, Lanzhou, 730000 China; 3 Department of Medical Education, Gansu Provincial Maternity and Childcare Hospital, Lanzhou, Gansu Province 730050 China

**Keywords:** Preterm birth, Methionine, Natural folate, Folic acid supplementation, Case control study

## Abstract

**Background:**

This study aimed to investigate the association of dietary methionine, natural folate and folic acid supplementation with the risk of preterm birth during pregnancy and to assess the interaction between dietary methionine and folic acid.

**Materials and methods:**

A case-control study conducted at Gansu Provincial Maternal and Child Health Hospital comprised 8,705 subjects, of whom 7,865 underwent normal delivery and 840 preterm birth. Eligible participants completed questionnaires to collect dietary and other baseline characteristic data. Unconditional logistic regression analysis was employed to assess the association between dietary methionine, natural folate and folic acid supplementation intake with preterm birth and its subtypes.

**Results:**

We found the risk of preterm birth was increased by 36% in the low dietary methionine intake group compared with the high group in late pregnancy (OR = 1.36, 95%CI: 1.10–1.70), and dietary methionine intake was significantly associated with medically induced preterm birth (*P*-trend = 0.0285). The risk of preterm birth was increased by 23% in the low natural folate intake group compared to the high group (OR = 1.23, 95%CI: 1.03–1.48) and natural folate intake was significantly associated with spontaneous preterm birth (*P*-trend = 0.0298). Those with low folic acid supplementation had a 105% increased risk of preterm birth (OR = 2.05, 95%CI: 1.15–3.65) and folic acid supplementation intake was significantly associated with spontaneous preterm birth (*P*-trend = 0.0195). Interaction analyses showed that the combination of low methionine and low natural folate further increased the risk (*P*-interaction < 0.0001). The combination of low methionine and low folate supplementation further increased the risk (*P*-interaction = 0.0005), and the risk of preterm birth was increased if intake of all three was low compared with the adequate intake group.

**Conclusion:**

Inadequate intake of dietary methionine, natural folate, and folic acid supplementation may elevate the risk of preterm birth. Moreover, low levels of these nutrients during pregnancy—specifically reduced methionine intake, insufficient natural folate intake, and inadequate folic acid supplementation—may act synergistically to further increase this risk. Attention should be paid to ensuring adequate intake of methionine and folic acid during late pregnancy and throughout gestation to reduce the incidence of preterm birth.

## Introduction

Preterm birth (PTB) refers to the delivery of a live infant prior to 37 completed weeks of gestation [1]. It affects nearly 15 million newborns each year worldwide and continues to be the predominant contributor to perinatal mortality [2]. Notably, the incidence of PTB in China has also shown an upward trend over the past decades, increasing from approximately 4%–5% in the 1990s to 7%–10% in recent years [3]. With the second-highest number of PTBs worldwide, PTB has become as a critical public health challenge in China.

Adequate nutritional intake during pregnancy is critical for optimizing maternal and fetal health [4]. The WHO recommends daily oral iron and folic acid supplementation as an evidence-based intervention to mitigate the risks of maternal anemia, low birth weight, and PTB [5].However, the average folic acid intake remains severely inadequate among pregnant women in Lanzhou, China [6]. Investigating the relationship between folic acid deficiency and PTB may facilitate the development of targeted nutritional strategies and interventions during pregnancy, thereby improving maternal and infant outcomes.

Recent studies have indicated that inadequate intake of one-carbon nutrients, particularly folic acid, is linked to adverse pregnancy outcomes, including PTB and low birth weight [7]. As an essential cofactor in the one-carbon metabolic pathway, folate is critical for nucleic acid synthesis, amino acid metabolism—such as the methionine cycle—cell proliferation, and DNA methylation [8]. Evidence from several investigations suggests that high-dose folic acid supplementation or elevated natural folate intake during pregnancy may reduce the risk of hypertensive disorders of pregnancy [9, 10], whereas insufficient folic acid intake has been associated with an increased likelihood of low-birth-weight infants [11]. Although physiological folate requirements rise substantially during pregnancy [12], dietary folate alone often does not meet these elevated demands. Therefore, this study examines both natural folate intake and supplemental folic acid within the context of maternal folate status.

Methionine is an essential amino acid and the starting amino acid for protein synthesis. Because transplacental transport is a primary pathway for delivering amino acids to the fetus, inadequate maternal methionine intake may impair fetal growth and development, thereby increasing the risk of PTB [13]. Folate metabolism is biochemically interconnected with the methionine cycle. Within one-carbon metabolism, folate is reduced to tetrahydrofolate (THF), while methionine is converted into S-adenosylmethionine (SAM), the universal methyl donor required for a broad range of methylation reactions [14, 15]. Both folate and methionine are central to DNA synthesis, repair and methylation [16], and functions that are particularly critical during early embryonic development when rapid cell division and epigenomic programming occur [17]. Despite these mechanistic links, the combined influence of natural dietary folate, folic acid supplementation, and methionine intake on PTB risk has not been thoroughly investigated. Evidence describing potential interactions among these nutrients remains limited, highlighting the need for further research.

Some cohort studies have reported that folate deficiency prior to conception and during pregnancy may elevate the risk of PTB [18, 19]. However, high-quality evidence directly examining the relationship between methionine intake and PTB is lacking, and the metabolic interplay between methionine and folic acid remains poorly understood. In addition, no studies have comprehensively assessed the combined effects of natural folate, methionine intake, and folic acid supplementation on PTB risk.

Therefore, the objective of this case control study was to evaluate the associations of natural folate intake, methionine intake, and folic acid supplementation during pregnancy with the risk of PTB, and to examine potential interactions among these three nutritional factors. The findings may provide a scientific basis for optimizing prenatal nutritional intervention strategies.

## Materials and methods

### Study population

A case control study was conducted over an 18-month period (January 2018 - June 2019) at the premier maternal and child health care facility in Lanzhou, Gansu Province. The study population comprised pregnant women aged ≥ 18 years without pre-existing psychiatric conditions who received regular antenatal care services. The research protocol received ethical approval from the Institutional Review Board of Gansu Maternal and Child Health Hospital (Approval No. 2018[029]), and all participants provided written informed consent prior to enrollment.

### Sample size calculation

Based on the baseline data from the preliminary cohort study, the study population consists of pregnant women who can be followed up and are willing to undergo regular prenatal check-ups at this hospital throughout their pregnancy and complete delivery (excluding pregnant women with delivery outcomes such as stillbirth, multiple births, miscarriage, or birth defects, as well as participants with missing data in gestational dietary questionnaires).

**Fig. 1:**
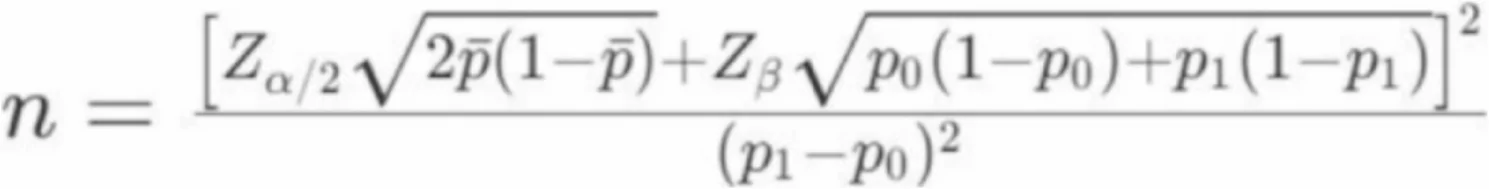
Sample size calculation formula

As illustrated in the sample size calculation formula (Fig. 1), the key parameters of this study were predefined based on previous published maternal nutritional cohort literature and preliminary pilot data: two-sided significance level (α) = 0.05, type II error rate (β) = 0.1 (corresponding to 90% statistical power). We set separate exposure prevalence values for three core nutritional indicators: low natural folate intake (control group, *p*_0_≈ 0.42; case group, *p*_1_ ≈ 0.58), low dietary methionine intake (control group, *p*_0_≈ 0.45; case group, *p*_1_ ≈ 0.61), and insufficient duration of folic acid supplementation (control group, *p*_0_ ≈ 0.30; case group, *p*_1_≈ 0.47). The effect size is reflected by the expected odds ratios (ORs) derived from the above exposure rates: OR = 1.90 for low natural folate intake, OR = 1.92 for low dietary methionine intake, and OR = 2.06 for insufficient folic acid supplementation. Using these parameters and the adopted formula, the largest minimum sample size required for a single indicator was calculated to be 203 participants for both the case group and the control group, resulting in a total minimum sample size of 406 participants. Our final analytical sample (840 preterm cases and 7,865 term controls) substantially exceeded this threshold, guaranteeing adequate statistical power to detect the hypothesized associations.

### Dietary survey

This study employed a validated semi-quantitative food frequency questionnaire (FFQ) administered through in-person interviews to assess dietary patterns and quantify daily intake of methionine, folic acid, and dietary supplements [20]. The comprehensive assessment protocol encompassed four domains: sociodemographic characteristics, medical records review, reproductive history documentation, and nutritional evaluation.

For precise dietary assessment, certified nutrition professionals conducted three consecutive 24-hour dietary recalls, capturing detailed consumption data across 12 major food categories. These included: (1) cereals, (2) fats/oils, (3) vegetables, (4) fruits, (5) poultry, (6) red meat/processed meats, (7) eggs, (8) aquatic products, (9) legumes/pulses, (10) dairy products, (11) mushrooms/seaweed, and (12) beverages/snacks, totaling 59 commonly consumed food items. All dietary quantification procedures followed standardized protocols using household measurement tools for portion size estimation.

This longitudinal assessment protocol implemented trimester-specific dietary evaluations conducted at three gestational phases: first trimester (weeks 1–13), second trimester (weeks 14–27), and third trimester pregnancy (> 27 weeks). Nutrient intake quantification utilized the reference-standard nutritional database from the Second Edition of China Food Composition (2009) following complete dietary data collection [21, 22]. Clinical documentation of obstetric complications and delivery parameters was systematically extracted from electronic health records. Case classification operationalized PTB as live births occurring before 37 completed weeks (< 259 days), with term deliveries (≥ 37 weeks) comprising the comparator group. PTB cases underwent clinical subclassification into iatrogenic medically indicate and spontaneous subtypes per standardized obstetric criteria.

### Covariate description

Socio-demographic characteristics included: maternal ethnicity (Han Chinese/Minority groups), age categorized as < 23 years, 23–35 years, or > 35 years, monthly household income (< 3,000 RMB vs. ≥3,000 RMB per capita), educational attainment (university degree or higher/below university), smoking exposure during pregnancy (active vs. passive) [23], and employment status (employed/unemployed). Active smoking was defined as self-reported tobacco use, while passive smoking was specified as daily exposure to environmental tobacco smoke for > 15 min.

Maternal health-related factors encompassed: pre-pregnancy body mass index (BMI, kg/m²), total gestational weight gain (kg), daily energy intake (kcal/day) during pregnancy, use of multivitamin supplements (yes/no), and obstetric complications including gestational diabetes mellitus (yes/no) and gestational hypertension (yes/no). Additional parameters included early-pregnancy anemia (hemoglobin < 110 g/L, diagnosed via venous blood assay; yes/no), history of miscarriage (yes/no), prior PTB (yes/no), and parity (primiparous/multiparous).

#### Definitions

Ethnicity was self-reported, income was defined as per capita household income, and anaemia was diagnosed based on standardized clinical criteria. Gestational complications were confirmed from medical records, and parity classification excluded pregnancies ending before 28 weeks.

### Statistical analysis

**Fig. 2:**
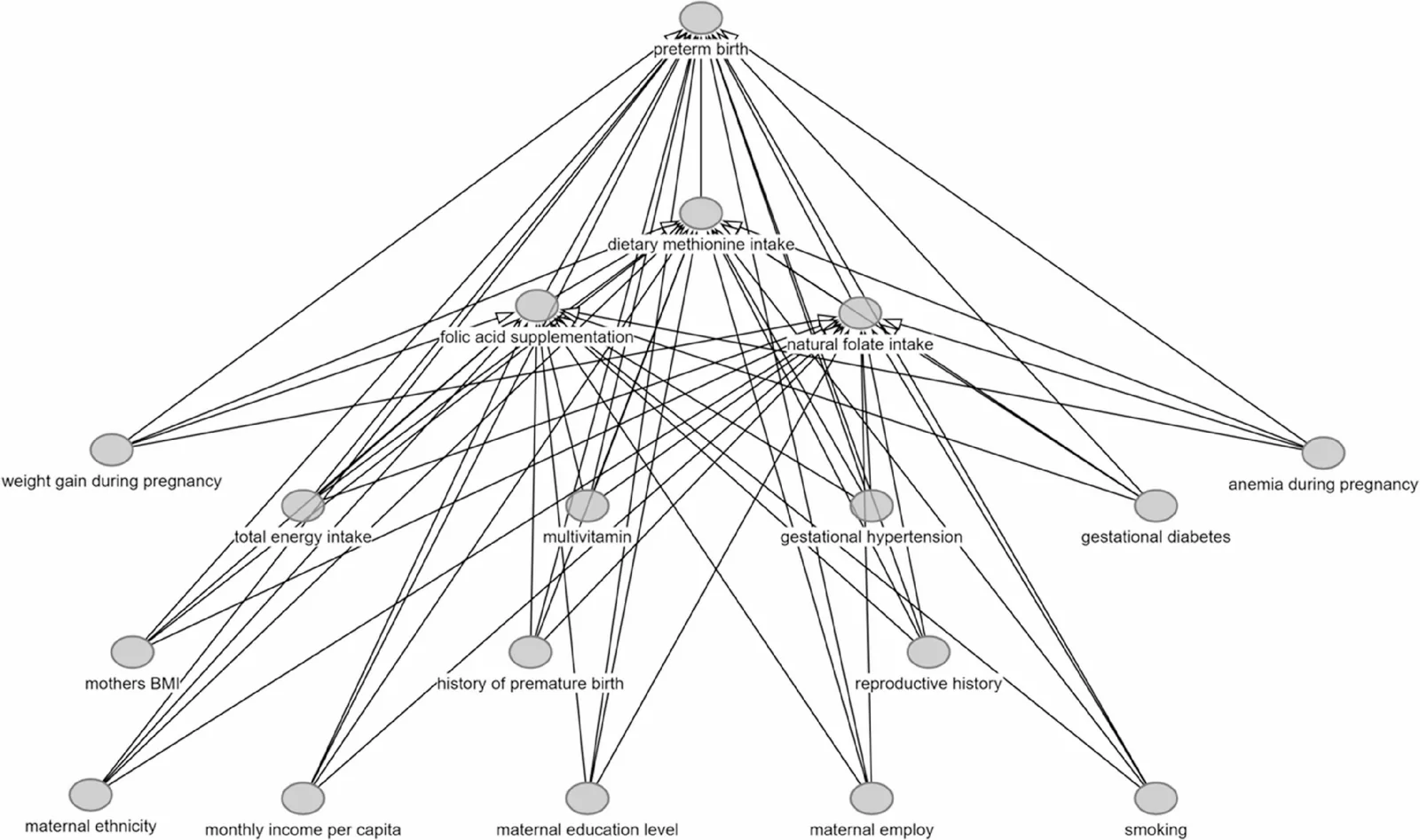
Directed acyclic graph identifying confounders for the associations between three one-carbon nutrient intakes and PTB

The chi-squared test was applied to compare frequency distributions of categorical variables between groups, and the Wilcoxon rank-sum test was used for intergroup comparisons of non-normally distributed continuous variables. Unconditional logistic regression was used to calculate odds ratios (ORs) and 95% confidence intervals (CIs) between dietary intake of methionine, folate, and folic acid supplementation and the risk of PTB and its clinical subtypes. We constructed a directed acyclic graph (DAG) to identify all potential backdoor confounding paths between folic acid supplementation, natural folate intake, dietary methionine intake and PTB. All confounders displayed in the DAG were incorporated into multivariate regression models to block confounding bias. The DAG is presented in Fig. 2.

As there are no defined reference values for methionine and recommended values for folic acid (dietary vs. supplemental) in the Chinese Dietary Reference Intakes (DRIs), this study used statistical software to statistically analyze the daily intake of vitamins and micronutrients during different trimesters based on obtaining critical values from the survey data. Dietary intake of methionine, folate was split into low and high subgroups using the optimal threshold identified by the maximum Youden index from ROC curve analysis. Supplemental folic acid exposure was measured as total supplementation weeks during gestation; we used the sample median of total weeks as the cut-off to divide participants into short-term and long-term supplementation groups. Dose–response linear trend analyses were subsequently conducted across graded intake levels, where *P* denotes the *P*-value for linear trend. This median stratization was an independent grouping scheme separate from the joint subgroups for interaction OR calculation. The association between the level of dietary methionine/folic acid intake and the risk of PTB may be non-linear. A multivariate adjusted cubic spline model (RCS) was used to explore the nonlinear association. The RCS model was adjusted for the above factors based on potential confounding. Multiplicative interaction parameters [OR = OR_11_/(OR_01_ × OR_10_)] and 95% CI were also estimated by including all variables [24]. All statistical tests were two-sided. Analyses were performed using SPSS software (version 23.0), SAS 9.4 (SAS Institute, Inc, Cary, NC, USA), with RCS modelling using R software version 4.4.2 (packages “Hmisc”, “rms”).

## Results

### Baseline characteristics of the study population

The analytic cohort comprised 8,705 eligible pregnancies following data quality control procedures, with 9.7% (*n* = 840) resulting in preterm deliveries and 90.3% (*n* = 7865) reaching term gestation. In Table 1, mothers who delivered preterm infants displayed distinct sociodemographic and clinical characteristics compared with those delivering at term. The preterm group had significantly lower socioeconomic status, reflected by reduced household income (*P* < 0.0001), lower educational attainment (*P* < 0.0001), and decreased workforce participation (*P* < 0.0001). Maternal ethnicity also differed between groups, with minority mothers more likely to deliver preterm infants (*P* = 0.0068). Maternal age also varied significantly across groups (*P* < 0.0001), with younger (<23 years) and older (>35 years) mothers presenting higher PTB proportions. Clinically, these mothers exhibited higher prepregnancy BMI (*P* = 0.0176), inadequate gestational weight gain (*P* < 0.0001), and a greater prevalence of hypertensive disorders (*P* < 0.0001). Behavioral risk factors were also more common in the preterm group, reflected by a higher incidence of tobacco exposure, including both active and passive smoking (*P* = 0.0139). Nutritional assessments indicated several differences, with this group reporting lower total energy intake (*P* < 0.0001), reduced adherence to micronutrient supplementation (multivitamins: *P* < 0.0001; folic acid: *P* < 0.0001), and lower dietary methionine (*P* < 0.0001) and folate intake (*P* < 0.0001). In addition, mothers with a history of premature birth and multiparous women showed significantly higher risk of PTB (both *P* < 0.0001). No statistically significant differences (*P* > 0.05) were identified between groups in the prevalence of gestational diabetes, early-pregnancy anemia status, prior miscarriage history, or neonatal sex distribution. These findings collectively outline the differential risk profiles associated with preterm delivery.

**Table 1:** Distributions of selected characteristics of the study population

Characteristics	Term(*n* = 7865)		Preterm (*n* = 840)		statistics	*P*-value
Total energy intake during pregnancy (kcal/d)	1686.65(1436.82,1970.63)		1554.35(1247.61,1831.11)			**<0.0001**
Folic acid supplementation during pregnancy					56.04	**< 0.0001**
≤ 8 Week	4227	(88.21)	565	(11.79)		
> 8 Week	3638	(92.97)	275	(7.03)		
multivitamin during pregnancy					16.85	**< 0.0001**
0Week	6150	(89.68)	708	(10.32)		
> 0 Week	1715	(92.85)	132	(7.15)		
Dietary methionine intake during pregnancy (mg/d)	1294.80 (1063.03,1571.68)		1169.97 (895.99,1438.83)			**<0.0001**
Natural folate intake during pregnancy(µg/d)	199.49 (154.14,262.23)		181.51 (134.73,235.94)			**<0.0001**
Maternal nation					7.33	**0.0068**
Han	7380	(90.57)	768	(9.43)		
Minority	485	(87.07)	72	(12.93)		
Maternal age					80.99	**< 0.0001**
< 23	487	(82.68)	102	(17.32)		
23–35	6684	(91.59)	614	(8.41)		
> 35	694	(84.84)	124	(15.16)		
Family monthly income per capita					75.05	**< 0.0001**
< 3000	4229	(87.88)	583	(12.12)		
≥ 3000	3636	(93.40)	257	(6.60)		
Maternal education level					122.71	**< 0.0001**
< College	2889	(85.93)	473	(14.07)		
≥ College	4976	(93.13)	367	(6.87)		
Maternal employ					40.11	**< 0.0001**
No	2331	(87.34)	338	(12.66)		
Yes	5534	(91.68)	502	(8.32)		
Pre-pregnancy BMI (kg/m^2^)					8.08	**0.0176**
< 18.5	1683	(90.87)	169	(9.13)		
18.5–24	5347	(90.60)	555	(9.40)		
> 24	835	(87.80)	116	(12.20)		
Weight gain during pregnancy(kg)					248.52	**< 0.0001**
< 15	2299	(83.12)	467	(16.88)		
15-18.5	2588	(92.79)	201	(7.21)		
> 18.5	2978	(94.54)	172	(5.46)		
Smoking (passive and active)					6.05	**0.0139**
No	6320	(90.74)	645	(9.26)		
Yes	1545	(88.79)	195	(11.21)		
Gestational diabetes					3.30	0.0692
No	7794	(90.41)	827	(9.59)		
Yes	71	(84.52)	13	(15.48)		
Gestational hypertension					249.67	**< 0.0001**
No	7582	(91.47)	707	(8.53)		
Yes	283	(68.03)	133	(31.97)		
Anemia during pregnancy					1.00	0.3169
No	6989	(90.24)	756	(9.76)		
Yes	876	(91.25)	84	(8.75)		
History of miscarriage					0.71	0.3988
No	6778	(90.46)	715	(9.54)		
Yes	1087	(89.96)	125	(10.31)		
History of premature birth					136.61	**< 0.0001**
No	7766	(90.85)	782	(9.15)		
Yes	99	(63.06)	58	(36.94)		
Reproductive history					61.53	**< 0.0001**
Primipara	5884	(91.84)	523	(8.16)		
Multiparous	1981	(86.21)	317	(13.79)		
Newborn’s sex					0.60	0.4164
Male	4135	(90.11)	454	(9.89)		
Female	3730	(90.62)	386	(9.38)		

Data are presented as median (interquartile range, IQR) for continuous nutrient intake variables; categorical variables are reported as n (%). Statistically significant results are shown in bold. For multivitamin use during pregnancy: “0 Week” means no multivitamin intake at any time in gestation; “>0 Week” refers to participants taking multivitamins for ≥ 1 week during pregnancy. For folic acid supplementation duration: The median total weeks of folic acid intake among all subjects was 8 weeks, which served as the grouping threshold. “≤8 weeks” and “>8 weeks” represent participants with folic acid supplementation duration below/above this median cutoff, respectively

### Relationship between dietary methionine, natural folate, and supplemental folic acid intake and risk of PTB during pregnancy

#### Low dietary methionine intake and higher PTB odds

In Table 2, following adjustment for potential confounding variables, the findings indicated that the likelihood of PTB was elevated in the third trimester among individuals with low methionine intake, as compared to those with higher intake. Low methionine intake during the third trimester was identified as a risk factor for PTB, with women in the low-intake group showing a higher odds of PTB compared with those with higher intake (OR^b^=1.36, 95% CI: 1.10–1.70). There was a dose-response correlation between increased methionine intake and the risk of PTB (*P* for trend = 0.0269).

Low methionine intake during the third trimester was also associated with a higher risk of medically indicated preterm birth, with the low-intake group showing increased odds both in late pregnancy (OR^b^ =1.78, 95% CI: 1.19–2.67) and across the entire gestational period (OR^b^ =1.76, 95% CI: 1.20–2.57).A dose-response pattern was observed, wherein higher methionine intake correlated with a lower risk of medically indicated PTB (*P* for trend < 0.05).

**Table 2:** Maternal dietary methionine intake categorized by cut-off value and the risk of Preterm

Dietary methionine intake	Term(*n *= 7865)	Preterm(*n *= 840)			Medically Indicated		Spontaneous	
(mg/day)			OR^a^(95%,CI)	OR^b^ (95% CI)	Cases	OR^b^ (95% CI)	Cases	OR^b^ (95%,CI)
First trimester								
Lower group	3470	484	1.72 (1.49,1.99)	1.09(0.89,1.33)	166	1.38(0.95,2.00)	318	1.01 (0.80,1.28)
High group	4395	356	1.00	1.00	96	1.00	260	1.00
Per 200-mg increase			0.85(0.82,0.89)	0.99(0.93,1.06)		0.96(0.85,1.08)		1.01(0.93,1.08)
* P* for trend			< 0.0001	0.8507		0.4497		0.8926
Second trimester								
Lower group	4025	549	1.80 (1.55,2.09)	1.20(0.98,1.47)	183	1.30 (0.89,1.90)	366	1.18 (0.94,1.50)
High group	3840	291	1.00	1.00	79	1.00	212	1.00
Per 200-mg increase			0.84(0.80,0.87)	0.96(0.90,1.03)		0.90(0.80,1.11)		0.99(0.92,1.07)
* P* for trend			< 0.0001	0.2296		0.0747		0.7970
Third trimester								
Lower group	1576	296	2.17 (1.87,2.53)	**1.36 (1.10**,**1.70)**	109	**1.78 (1.19**,**2.67)**	187	1.27(0.98,1.64)
High group	6289	544	1.00	1.00	153	1.00	391	1.00
Per 200-mg increase			0.82(0.79,0.86)	**0.93(0.88**,**0.99)**		**0.88(0.78**,**0.99)**		0.95(0.89,1.02)
* P* for trend			< 0.0001	0.0269		0.0285		0.1823
During pregnancy								
Lower group	3765	529	1.85(1.60,2.15)	1.20(0.97,1.47)	185	**1.76(1.20**,**2.57)**	344	1.04 (0.82,1.33)
High group	4100	311	1.00	1.00	77	1.00	234	1.00
Per 200-mg increase			0.82(0.78,0.85)	0.94(0.88,1.00)		**0.87(0.77**,**0.98)**		0.96(0.89,1.04)
* P* for trend			< 0.0001	0.0515		0.0208		0.3504

OR^a^univariate analyses.OR^b^adjusted for maternal ethnicity, weight gain during pregnancy, mother’s BMI, total energy intake, monthly income per capita, maternal education level, smoking, maternal employ, multivitamin, folic acid supplementation, gestational hypertension, gestational diabetes, anemia during pregnancy, history of premature birth, reproductive history and natural folate intake. *P* for trend based on OR^b^ adjusted for Per 200-mg increase. Total preterm birth includes two clinical subtypes: spontaneous (labor onset without medical intervention) and medically indicated (delivery initiated medically for maternal or fetal indications). Values in bold font denote statistically significant associations, defined as 95% CIs for adjusted odds ratios that do not cross 1

#### Low natural folate intake and higher PTB odds

In Table 3, low natural folate intake during the third trimester was associated with an elevated risk of PTB, with the low-intake group showing a 23% increase in risk compared with the high-intake group (OR^b^=1.23, 95% CI: 1.03–1.48). Moreover, a dose-response trend was also evident, indicating that higher natural folate intake corresponded to a lower likelihood of PTB (*P* for trend = 0.0301).

Further analysis by PTB subtype showed that low natural folate intake in the third trimester was linked to a 26% higher risk of spontaneous PTB compared with the high-intake group (OR^b^=1.26, 95% CI: 1.02–1.56), again demonstrating a significant dose–response relationship (*P* for trend = 0.0298). Similar associations were observed when natural folate intake was assessed across the entire pregnancy.

**Table 3:** Maternal natural folate intake categorized by cut-off value and the risk of Preterm

Natural folate intake	Term(*n *= 7865)	Preterm(*n *= 840)			Medically Indicated		Spontaneous	
(µg/day)			OR^a^ (95%,CI)	OR^b^ (95%,CI)	Cases	OR^b^ (95%,CI)	Cases	OR^b^ (95%,CI)
First trimester								
Lower group	3677	475	1.48(1.28,1.71)	1.18(0.99,1.39)	154	1.11(0.80,1.52)	321	1.19 (0.98,1.46)
High group	4188	365	1.00	1.00	108	1.00	257	1.00
Per 50-µg increase			0.85(0.81,0.90)	0.94(0.88,1.00)		0.95(0.84,1.08)		0.94(0.87,1.01)
* P* for trend			< 0.0001	0.0659		0.4447		0.0929
Second trimester								
Lower group	1993	295	1.60(1.37,1.85)	1.17(0.97,1.40)	96	1.11(0.78,1.56)	199	1.17(0.94,1.45)
High group	5872	545	1.00	1.00	166	1.00	379	1.00
Per 50-ug increase			0.86(0.82,0.91)	0.95(0.89,1.02)		0.96(0.85,1.10)		0.96(0.88,1.03)
* P* for trend			< 0.0001	0.1363		0.5666		0.2437
Third trimester								
Lower group	2309	344	1.67(1.44,1.93)	**1.23(1.03**,**1.48)**	110	1.10 (0.79,1.54)	234	**1.26 (1.02**,**1.56)**
High group	5556	496	1.00	1.00	152	1.00	344	1.00
Per 50-µg increase			0.84(0.80,0.89)	**0.93(0.87**,**0.99)**		0.98(0.87,1.10)		**0.92(0.85**,**0.99)**
* P* for trend			< 0.0001	0.0301		0.7234		0.0298
During pregnancy								
Lower group	1986	308	1.71(1.48,1.99)	**1.27 (1.05**,**1.53)**	101	1.15 (0.82,1.62)	207	**1.30 (1.05**,**1.62)**
High group	5879	532	1.00	1.00	161	1.00	371	1.00
Per 50-µg increase			0.84(0.80,0.89)	**0.93(0.87**,**0.99)**		0.99(0.87,1.12)		**0.91(0.84**,**0.99)**
* P* for trend			< 0.0001	0.0333		0.8697		0.0252

OR^a^ univariate analyses. OR^b^ adjusted for maternal ethnicity, weight gain during pregnancy, mother’s BMI, total energy intake, monthly income per capita, maternal education level, smoking, maternal employ, multivitamin, folic acid supplementation, gestational hypertension, history of premature birth, gestational diabetes, anemia during pregnancy, reproductive history and dietary methionine intake. *P*-value trends based on OR^b^ adjusted for factor with each 50 µg decreased. Definitions of preterm subtypes and bold significance markers are consistent with the footnote of Table 2

#### Dietary intake of methionine and folate within a certain range and PTB odds

**Fig. 3:**
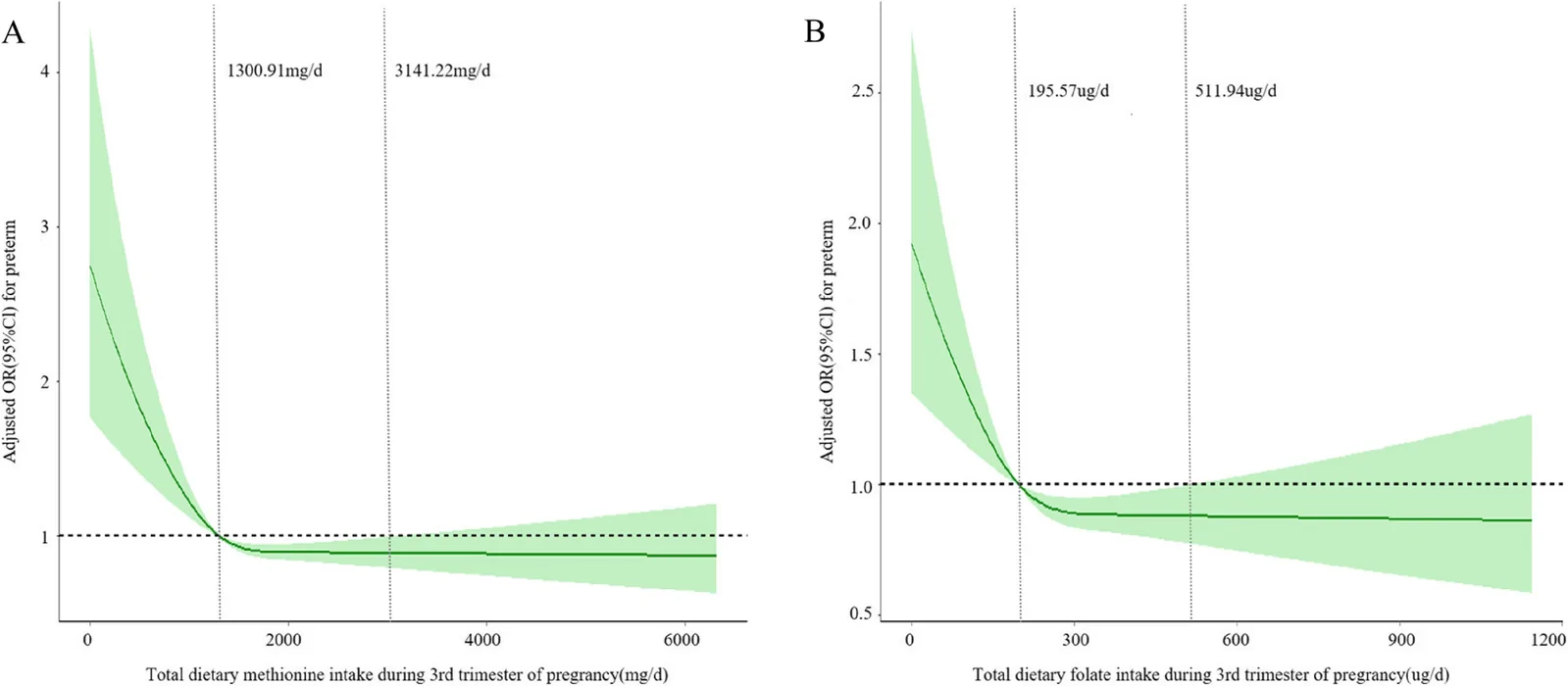
Restricted cubic spline models of Preterm risk associated with methionine intake at the third trimester (**A**), folate intake at the third trimester (**B**)

As shown in Fig. 3, a non-linear association was observed between dietary methionine intake and the risk of PTB in the third trimester. When intake increased from 0 to 1300.91 mg/day, the risk of PTB decreased correspondingly (*P* non-linear < 0.05). However, as methionine intake continued to rise within the range of 1300.908- 3141.22 mg/day higher intake was associated with a reduced risk of PTB (*P* non-linear < 0.05).

A similar non-linear pattern emerged for natural folate. In the third trimester, natural folate intake between 0 and 195.57 ug/day was associated with an increased risk of PTB (*P* non-linear < 0.05). Beyond this level, from 195.57 to 511.94 ug/day, increasing natural folate intake continued to reduce the risk of PTB (*P* non-linear < 0.05).

#### Low folic acid supplementation intake and higher PTB odds

In Table 4, low folic acid supplementation intake in the third trimester was associated with a higher risk of PTB compared with higher intake (OR^b^ =2.05, 95% CI 1.15–3.65). When PTB subtypes were examined, low folic acid supplementation similarly increased the risk of spontaneous PTB (OR^b^=2.34, 95% CI 1.14–4.80). A clear dose–response pattern was observed, whereby increasing folic acid supplementation intake was associated with a reduced risk of third-trimester PTB (*P* for trend = 0.0083) and Spontaneous PTB (*P* for trend = 0.0195). The first trimester and throughout the pregnancy produced similar outcomes to those of the third trimester.

**Table 4:** Maternal folic acid supplementation categorized by cut-off value and the risk of Preterm

Folic acid supplementation	Term(*n *= 7865)	Preterm(*n *= 840)			Medically Indicated		Spontaneous	
(week)			OR^a^(95%,CI)	OR^b^ (95%,CI)	Cases	OR^b^ (95%,CI)	Cases	OR^b^ (95%,CI)
First trimester								
Lower group	4484	586	1.74 (1.49,2.03)	**1.30 (1.10**,**1.54)**	181	0.94(0.69,1.29)	405	**1.48(1.21**,**1.79)**
High group	3381	254	1.00	1.00	81	1.00	173	1.00
Per 1-week increase			0.96(0.94,0.97)	**0.98(0.97**, 1.00)*		1.02 (1.00,1.05)		**0.97(0.95**,**0.99)**
* P* for trend			< 0.0001	0.0318		0.0949		0.0002
Second trimester								
Lower group	7233	783	1.20 (0.91,1.59)	1.09(0.81,1.46)	251	1.55(0.81,2.97)	532	0.94 (0.68,1.31)
High group	632	57	1.00	1.00	11	1.00	46	1.00
Per 1-week increase			0.99(0.96,1.02)	1.00(0.97,1.03)		0.97(0.91,1.03)		1.01(0.98,1.04)
* P* for trend			0.3874	0.7496		0.3279		0.6203
Third trimester								
Lower group	7629	827	1.97(1.12,3.46)	**2.05 (1.15**,**3.65)**	257	1.49(0.58,3.78)	570	**2.34(1.14**,**4.80)**
High group	236	13	1.00	1.00	5	1.00	8	1.00
Per 1-week increase			0.90(0.83,0.98)	**0.90(0.83**,**0.97)**		0.93 (0.82,1.06)		**0.89(0.80**,**0.98)**
* P* for trend			0.0110	0.0083		0.2713		0.0195
During pregnancy								
Lower group	4227	565	1.77(1.52,2.06)	**1.33 (1.13**,**1.57)**	177	1.04(0.76,1.40)	388	**1.46 (1.21**,**1.77)**
High group	3638	275	1.00	1.00	85	1.00	190	1.00
Per 1-week increase			0.96(0.95,0.97)	**0.98 (0.97**,1.00)*		1.007 (0.99,1.03)		**0.974(0.96**,**0.99)**
* P* for trend			< 0.0001	0.0093		0.5091		0.0006

OR^a^ univariate analyses. OR^b^ adjusted for maternal ethnicity, weight gain during pregnancy, mother’s BMI, total energy intake, monthly income per capita, maternal education level, smoking, maternal employ, multivitamin, natural folate intake, gestational hypertension, history of premature birth, gestational diabetes, anemia during pregnancy, reproductive history and dietary methionine intake. *P* for trend based on Per 1-week increase. The symbol * indicates that the 95% CI is rounded to 1.00, but the exact upper limit is < 1 (i.e., actually significant). Definitions of preterm subtypes and bold significance markers are consistent with the footnote of Table 2

### Interaction of maternal intake with the risk of PTB

#### Interaction of maternal intake of methionine and natural folate with the risk of PTB

In Table 5, concurrent low dietary methionine and natural folate intake in the third trimester was associated with a substantially higher risk of PTB, yielding a 57% increase in risk compared with women who had adequate intakes (adjusted interaction odds ratio OR^f^ =1.57, 95% CI: 1.27–1.95). This synergistic effect remained evident across the entire pregnancy, with dual-deficient participants experiencing a 38% higher PTB risk (OR^f^=1.38, 95% CI: 1.13–1.67).

**Table 5:** Interaction effects of maternal dietary methionine and natural folate intake on the risk of Preterm

Dietary methionine intake	Natural folate intake	Lower group		High group
	Cases	OR^b^ (95%,CI)	Cases	OR^b^ (95%,CI)
First trimester				
Lower group	365/2411	1.05(0.97,1.14)	119/1059	1.13 (0.84,1.52)
High group	110/1266	1.04(0.91,1.17)	246/3129	1.00
	OR^f^ (95%,CI) = 1.22 (1.02,1.47) *P* = 0.0300			
Second trimester				
Lower group	281/1782	1.09(0.98,1.21)	268/2243	1.18(0.94,1.48)
High group	14/211	0.87 (0.65,1.18)	277/3629	1.00
	OR^f^ (95%,CI) = 1.27(1.06,1.54) *P* = 0.0119			
Third trimester				
Lower group	220/1053	**1.18(1.07**,**1.29)**	76/523	1.17(0.84,1.64)
High group	124/1256	1.05 (0.94,1.18)	420/5033	1.00
	OR^f^ (95%,CI) = 1.57(1.27,1.95) *P* < 0.0001			
During pregnancy				
Lower group	287/1725	**1.14(1.02**,**1.26)**	242/2040	1.23(0.98,1.55)
High group	21/261	0.95 (0.74,1.22)	290/3839	1.00

OR^b^ adjusted for maternal ethnicity, weight gain during pregnancy, mother’s BMI, total energy intake, monthly income per capita, maternal education level, smoking, maternal employ, multivitamin, gestational hypertension, history of premature birth, reproductive history and folic acid supplementation. OR^f^ as the odds ratio calculated from the multiplicative interaction model and bold significance markers are consistent with the footnote of Table 2. OR^f^ (95%,CI) = 1.38(1.13,1.67) *P* = 0.0012

#### Interaction of maternal intake of methionine and folic acid supplementation with the risk of PTB

In Table 6, concurrent low dietary methionine intake and low folic acid supplementation in the first trimester was associated with a significantly higher risk of PTB, resulting in a 38% increased risk compared with women with adequate intake levels (OR^f^=1.38, 95% CI: 1.15–1.65). This synergistic interaction persisted across pregnancy, with elevated PTB risks observed in the second trimester (OR^f^ = 1.41, 95% CI: 1.18–1.68), third trimester (OR^f^ = 1.47, 95% CI: 1.18–1.82), and throughout pregnancy (OR^f^ = 1.43, 95% CI: 1.19–1.71).

**Table 6:** Interaction effects of maternal dietary methionine intake and folic acid supplementation on the risk of Preterm

Dietary methionine intake	Folic acid supplementation	Lower group		High group
	Cases	OR^b^ (95%,CI)	Cases	OR^b^ (95%,CI)
First trimester				
Lower group	357/2052	**1.12(1.02**,**1.23)**	127/1418	0.93(0.66,1.30)
High group	208/2175	1.11(0.98,1.24)	148/2220	1.00
OR^f^ (95%,CI) = 1.38(1.15,1.65) *P* = 0.0006				
Second trimester				
Lower group	392/2325	**1.14 (1.04**,**1.26)**	157/1700	1.14(0.82,1.58)
High group	173/1902	1.10 (0.97,1.25)	118/1938	1.00
OR^f^ (95%,CI) = 1.41(1.18,1.68) *P* = 0.0001				
Third trimester				
Lower group	227/1032	**1.22 (1.10**,**1.37)**	69/544	1.41(0.94,2.11)
High group	338/3195	1.15 (1.05,1.27)	206/3094	1.00
OR^f^ (95%,CI) = 1.47(1.18,1.82) *P* = 0.0005				
During pregnancy				
Lower group	385/2223	**1.16 (1.06**,**1.28)**	144/1542	1.03(0.73,1.46)
High group	180/2004	1.10(0.97,1.24)	131/2096	1.00

OR^b^ adjusted for maternal ethnicity, weight gain during pregnancy, mother’s BMI, total energy intake, monthly income per capita, maternal education level, smoking, maternal employ, multivitamin, natural folate intake, gestational hypertension, history of premature birth and reproductive history. OR^f^ as the odds ratio calculated from the multiplicative interaction model and bold significance markers are consistent with the footnote of Table 2. OR^f^ (95%,CI) = 1.43(1.19,1.71) *P*= 0.0001

#### Interaction between maternal intake of methionine, natural folate and folic acid supplementation and risk of PTB.

In Table 7, we further assessed the joint effects of low maternal intake of methionine, natural folate, and folic acid supplements on PTB risk. In the third trimester, women with concurrently low intake of all three nutrients showed a significantly elevated risk of PTB, with a 12% increase compared with those with adequate intake levels (OR^b^ = 1.12, 95% CI: 1.05–1.19). A similar increased risk was observed across the entire pregnancy period (OR^b^ = 1.09, 95% CI: 1.02–1.16). Consistent patterns were also detected in the first and second trimesters, indicating that combined deficiencies in methionine, natural folate, and supplemental folic acid throughout gestation contribute to a higher likelihood of PTB.

**Table 7:** Interaction effects of maternal dietary methionine intake、folic acid supplementation and natural folate intake on the risk of Preterm

Dietary methionine intake	Low Folic acid supplementation and low Natural folate intake		Low Folic acid supplementation and high Natural folate intake		High Folic acid supplementation and low Natural folate intake		High Folic acid supplementation and high Natural folate intake	
	Cases	OR^b^ (95%,CI)	Cases	OR^b^ (95%,CI)	Cases	OR^b^ (95%,CI)	Cases	OR^b^ (95%,CI)
First trimester								
Lower group	276/1454	**1.07(1.02**,**1.12)**	81/598	**1.10 (1.01**,**1.19)**	89/957	0.99(0.86,1.13)	38/461	1.04(0.63,1.72)
High group	63/601	1.04 (0.98,1.11)	145/1574	1.05 (0.98,1.13)	47/665	1.02(0.84,1.23)	101/1555	1.00
Second trimester								
Lower group	214/1085	**1.08(1.01**,**1.14)**	178/1240	**1.07(1.01**,**1.15)**	67/697	0.98 (0.83,1.17)	90/1003	1.09(0.76,1.57)
High group	8/105	0.89(0.77,1.03)	165/1797	1.06(0.99,1.13)	6/106	0.93(0.59,1.47)	112/1832	1.00
Third trimester								
Lower group	171/698	**1.12(1.05**,**1.19)**	56/334	1.08(0.98,1.20)	49/355	1.12(0.94,1.33)	20/189	1.24 (0.66,2.33)
High group	83/644	**1.07(1.01**,**1.13)**	255/2551	**1.06(1.00**,1.12)*	41/612	0.97 (0.80,1.18)	165/2482	1.00
During pregnancy								
Lower group	213/1061	**1.09(1.02**,**1.16)**	172/1162	**1.09(1.02**,**1.16)**	74/664	1.04(0.87,1.24)	70/878	1.01 (0.69,1.50)
High group	13/118	0.99(0.88,1.11)	167/1886	1.05(0.98,1.11)	8/143	0.86(0.57,1.28)	123/1953	1.00

OR^b^ adjusted for maternal ethnicity, weight gain during pregnancy, mother’s BMI, total energy intake, monthly income per capita, maternal education level, smoking, maternal employ, multivitamin, gestational hypertension, history of premature birth and reproductive history. The symbol * indicates that the 95% CI is rounded to 1.00, but the exact upper limit is > 1 (i.e., actually significant) and bold significance markers are consistent with the footnote of Table 2

## Discussion

This study demonstrates that isolated deficiencies in natural folate, methionine, or folic acid supplementation during pregnancy—particularly in the third trimester—significantly elevate the risk of PTB. Deficiency in natural folate or inadequate folic acid supplementation during pregnancy was significantly associated with an increased risk of spontaneous PTB, whereas methionine deficiency was more strongly associated with medically indicated PTB. A significant dose–response association was observed between natural folate and methionine intake levels and PTB risk. Notably, concurrent exposure to low natural folate and low methionine in late pregnancy was associated with a 1.57-fold higher risk of PTB relative to controls, accompanied by a significant multiplicative interaction (*P* < 0.0001), suggesting a potential synergistic contribution of these nutrients to methyl-donor metabolism. In addition, the stronger synergistic effect between inadequate methionine intake and folic acid supplementation—compared with low natural folate intake—may reflect the substantially greater bioavailability of supplemental folic acid (≈ 85%) relative to natural folate (≈ 50%).

To date, only a limited number of studies have examined the combined effects of natural folate, methionine, and folic acid supplementation on PTB across different trimesters [25]. However, the existing findings remain inconsistent. For instance, several studies reported no significant association between periconceptional natural folate or folic acid supplementation and PTB [26]. Studies by Wang, Du, and colleagues found that pregnant women who experienced PTB had significantly lower serum folate concentrations compared with those with term deliveries [27, 28], and that folate deficiency during pregnancy was associated with an elevated risk of PTB [29]. Additionally, Mao et al. demonstrated that folic acid supplementation and natural folate intake during pregnancy exert synergistic protective effects against PTB and certain PTB subtypes [30]. The findings of the present study are consistent with this body of evidence.

Perinatal folic acid supplementation is well established to reduce the risk of neural tube defects in newborns [31]. In colorectal cancer treatment, folic acid supplementation has been shown to be more effective than natural folate, with its efficacy influenced by methionine status and methylenetetrahydrofolate reductase (MTHFR) polymorphisms [32]. Although these findings are not directly related to PTB, they highlight the importance of considering upper intake limits for folic acid and its potential metabolic interactions. Moreover, attention should be given to MTHFR genetic polymorphisms, which may modify the effects of folate and methionine intake during pregnancy and, in turn, influence fetal development [33–35].

This study provides a comprehensive evaluation of the effects of natural folate, methionine, and folic acid supplementation on PTB. Clinical phenotyping of PTB in this cohort indicated that low intakes of natural folate, methionine, or folic acid supplementation were associated with an increased risk of PTB. Given the expanding evidence on the combined effects of folate, methionine, and folic acid supplementation on PTB, further studies are warranted to validate and elucidate these associations.

Based on one-carbon metabolism of folate and methionine, we propose the “methyl donor depletion–placental dysfunction” hypothesis. Folate deficiency reduces 5-methyltetrahydrofolate (5-MTHF) levels, thereby impairing homocysteine (Hcy) remethylation in the methionine cycle and inducing hyperhomocysteinemia [36]. Accumulation of Hcy can damage vascular endothelial cells, impair trophoblast function, and promote the release of pro-inflammatory factors, ultimately resulting in inadequate placental perfusion [37]. Maternal folate deficiency also inhibits placental mTOR signaling and reduces amino acid transporter activity, thereby impairing fetal development [38]. These mechanisms may contribute to the pathogenesis of PTB.

In addition, methionine and folate, as primary sources of methyl donors, when deficient, further exacerbate methyl donor depletion and impair DNA repair, collectively impacting fetal development [39].

This study evaluates both dietary intake and supplementation to assess PTB risk, thereby addressing limitations of previous studies that focused on a single nutrient source. Moreover, precise gestational age assessment and detailed clinical phenotyping of PTB provide valuable evidence on the relationships between natural folate, methionine, and supplemental folate intake and PTB across different gestational periods.

Nevertheless, several limitations should be acknowledged. First, data on natural folate, methionine, and supplemental folate intake were self-reported, making recall bias unavoidable. Second, pre-pregnancy folate and methionine intake were not recorded, which may have led to an underestimation of the impact of maternal nutrient reserves on pregnancy outcomes. Third, although interactions between dietary multivitamins and micronutrients could not be fully quantified, potentially introducing confounding, rigorous quality control measures were implemented from questionnaire design to data entry to minimize recall bias.

Conversely, detailed demographic, medical history, and lifestyle data allowed adjustment for confounders, and PTB diagnoses, including subtypes, were based on medical records rather than self-report, reducing the risk of misclassification. Nonetheless, residual unmeasured confounding and the inability to establish causality cannot be fully excluded. Moreover, the mechanistic role of methionine has not been fully validated, and future studies should integrate dietary, supplementation, and metabolic biomarkers to develop multidimensional intervention models. Nonetheless, this represents the first large-scale study to systematically and comprehensively investigate the synergistic effects of natural folate, methionine, and supplemental folate intake on PTB, providing a valuable framework for future research.

Furthermore, we performed separate regression models to evaluate trimester-specific associations between methionine, folate, folic acid supplementation intake and PTB, instead of a single integrated model with mutual adjustment for nutrient intake across all gestational trimesters. Dietary patterns remained stable during pregnancy: participants with higher nutrient intake in one trimester generally had elevated intake in the other two trimesters. Concurrent inclusion of intake levels from all three trimesters in one model would introduce severe multicollinearity and yield unstable regression coefficients, so we chose to fit independent trimester models. However, as reported by Wilson et al. [40], this analytical strategy may bias the identification of critical exposure windows given the absence of cross-trimester adjustment. Therefore, our trimester-specific findings should be interpreted cautiously, and future studies adopting statistical approaches to mitigate multicollinearity are warranted to construct mutually adjusted models and draw more reliable conclusions on critical windows.

## Conclusion

In conclusion, our study demonstrates that insufficient dietary intake of natural folate, methionine, and folic acid supplementation during pregnancy, particularly in the late stages of gestation, is significantly associated with an increased risk of PTB, and the low intake of these nutrients may have a synergistic effect in elevating PTB risk. Promotion of adequate natural folate, methionine, and folic acid supplementation during pregnancy, especially in late gestation, could help reduce the incidence of PTB in regions such as Lanzhou, Gansu Province, Northwest China. To validate these findings and elucidate the underlying mechanisms, future studies should integrate data on maternal intake of other one-carbon–related nutrients, relevant biomarkers, genetic polymorphisms, and environmental factors.

## Data Availability

The datasets generated and/or analysed during the current study are not publicly available due to data confidentiality principles, but are available from the corresponding author on reasonable request.

## Abbreviations

OR: Odds ratio
95% CI: 95% confidence interval
PTB: Preterm birth
FFQ: Food frequency questionnaire
BMI: Body mass index

## References

[1] Ohuma EO, Moller AB, Bradley E, Chakwera S, Hussain-Alkhateeb L, Lewin A, Okwaraji YB, Mahanani WR, Johansson EW, Lavin T, et al. National, regional, and global estimates of preterm birth in 2020, with trends from 2010: a systematic analysis. Lancet. 2023;402(10409):1261–71. 10.1016/S0140-6736(23)00878-4 37805217

[2] Liu L, Oza S, Hogan D, Perin J, Rudan I, Lawn JE, Cousens S, Mathers C, Black RE. Global, regional, and national causes of child mortality in 2000-13, with projections to inform post-2015 priorities: an updated systematic analysis. Lancet. 2015;385(9966):430–40. 10.1016/S0140-6736(14)61698-6 25280870

[3] Zhang Y, Zhu Y, Chen C. Prematurity incidence and trends. Chin J Neonatology. 2021;36(4):74–7.

[4] Lassi ZS, Padhani ZA, Rabbani A, Rind F, Salam RA, Bhutta ZA. Effects of nutritional interventions during pregnancy on birth, child health and development outcomes: A systematic review of evidence from low- and middle-income countries. Campbell Syst Rev. 2021;17(2):e1150. 10.1002/cl2.1150 PMC8356342 37131924

[5] Garcia-Casal MN, Estevez D, De-Regil LM. Multiple micronutrient supplements in pregnancy: Implementation considerations for integration as part of quality services in routine antenatal care. Objectives, results, and conclusions of the meeting. Matern Child Nutr. 2018;14(Suppl 5):e12704. 10.1111/mcn.12704 PMC6866095 30585705

[6] Mao B, Wang Y, Li J, Wang W, Wang J, Xu X, Zhou M. Investigation and evaluation on dietary intake of vitamins and microelements among pregnant women in urban areas of Lanzhou. Chin J Women Child Health. 2020;11(01):10–6.

[7] Guo H, Mao B, Wang M, Liu Q, Yang L, Xie Y, Wang Y, He X, Cui H, Lin X, et al. Folic acid supplementation, dietary folate intake and risk of small for gestational age in China. Public Health Nutr. 2020;23(11):1965–73. 10.1017/S1368980019003331 PMC10200399 31787119

[8] Shulpekova Y, Nechaev V, Kardasheva S, Sedova A, Kurbatova A, Bueverova E, Kopylov A, Malsagova K, Dlamini JC, Ivashkin V. The concept of folic acid in health and disease. Molecules. 2021;26(12):3731. 10.3390/molecules26123731. PMC8235569 34207319

[9] Liu X, Mao B, Dong Y, Liu X, Liu X. Study on correlation between folic acid intake of pregnant women in perigestational period and HDCP. Chin J Woman Child Health Res. 2020;31(09):1209–17.

[10] Mao B, Qiu W, Wang Y, Wang J, et al. Study on the correlation between folic acid supplementation before and during preg-nancy and hypertensive disorder complicating pregnancy. Maternal Child Health Care China. 2017;32(24):6087–91.

[11] Mao B, Qiu W, Wang Y, Li J, Wang J, Wang W, et al. Study on the correlation between maternal folic acid intake and low birth weight in-fant. Maternal Child Health Care China. 2018;33(16):3772–6.

[12] Rondo PH, Tomkins AM. Folate and intrauterine growth retardation. Ann Trop Paediatr. 2000;20(4):253–8. 10.1080/02724936.2000.11748144 11219161

[13] Cai S, Ye Q, Zeng X. Effects and Mechanism of Methionine on Reproductive Performance of Livestock and Poultry. Chin J Anim Nutr. 2018;30(3):881–7.

[14] Sanderson SM, Gao X, Dai Z, Locasale JW. Methionine metabolism in health and cancer: a nexus of diet and precision medicine. Nat Rev Cancer. 2019;19(11):625–37. 10.1038/s41568-019-0187-8 31515518

[15] Sfakianoudis K, Zikopoulos A, Grigoriadis S, Seretis N, Maziotis E, Anifandis G, Xystra P, Kostoulas C, Giougli U, Pantos K, et al. The role of one-carbon metabolism and methyl donors in medically assisted reproduction: a narrative review of the literature. Int J Mol Sci. 2024;25(9):4977. 10.3390/ijms25094977. PMC11084717 38732193

[16] Yang C, Zhang J, Liao M, Yang Y, Wang Y, Yuan Y, Ouyang L. Folate-mediated one-carbon metabolism: a targeting strategy in cancer therapy. Drug Discov Today. 2021;26(3):817–25. 10.1016/j.drudis.2020.12.006 33316375

[17] Sapehia D, Mahajan A, Srinivasan R, Kaur J. Pre-natal dietary imbalance of folic acid and vitamin B12 deficiency adversely impacts placental development and fetal growth. Placenta. 2023;132:44–54. 10.1016/j.placenta.2023.01.003 36657272

[18] Ye C, Chen S, Wang T, Zhang S, Qin J, Chen L. Risk factors for preterm birth: a prospective cohort study. Zhongguo Dang Dai Er Ke Za Zhi. 2021;23(12):1242–9. 10.7499/j.issn.1008-8830.2108015 PMC8690713 34911607

[19] Bekele Y, Gallagher C, Batra M, Vicendese D, Buultjens M, Erbas B. Is oral iron and folate supplementation during pregnancy protective against low birth weight and preterm birth in Africa? a systematic review and meta-analysis. Nutrients. 2024;16(16):2801. 10.3390/nu16162801. PMC11356927 39203937

[20] Wang Y, Zhao N, Qiu J, He X, Zhou M, Cui H, Lv L, Lin X, Zhang C, Zhang H, et al. Folic acid supplementation and dietary folate intake, and risk of preeclampsia. Eur J Clin Nutr. 2015;69(10):1145–50. 10.1038/ejcn.2014.295 PMC4517985 25626412

[21] Yang Y, Wang G, Pan X. China Food Composition. Beijing: Peking University Medical Press; 2009.

[22] Guo C, Zhai F, Lin X, et al. Chinese dietary reference intakes—Part 5: Water-soluble vitam. WS/T 578.5–2018. Beijing: National health commission of the people’s republic of China. 2018. https://www.nhc.gov.cn/ewebeditor/uploadfile/2018/05/20180516113333796.pdf.

[23] Qiu J, He X, Cui H, Zhang C, Zhang H, Dang Y, Han X, Chen Y, Tang Z, Zhang H, et al. Passive smoking and preterm birth in urban China. Am J Epidemiol. 2014;180(1):94–102. 10.1093/aje/kwu092 PMC4070933 24838804

[24] Andersson T, Alfredsson L, Källberg H, Zdravkovic S, Ahlbom A. Calculating measures of biological interaction. Eur J Epidemiol. 2005;20(7):575–9. 10.1007/s10654-005-7835-x 16119429

[25] Vidmar Golja M, Trontelj J, Geršak K, Mlinarič-Raščan I, Šmid A. Simultaneous quantification of intracellular concentrations of clinically important metabolites of folate-homocysteine cycle by LC-MS/MS. Anal Biochem. 2020;605:113830. 10.1016/j.ab.2020.113830 32717185

[26] Bai Y, Liu C, Zhang J, Meng W, et al. Association between periconceptional supplementation of folic acid or multiple ⁃micro⁃nutrients containing folic acid and preterm delivery in women. J Peking Univ (health sciences). 2023;55(03):495–501. 10.19723/j.issn.1671-167X.2023.03.016 PMC10258057 37291926

[27] Du M, Jin C, Gu Z. Relationship between polymorphism of key gene locus of folic acid metabolism and premature deliveryin pregnant women. J Xinxiang Med Univ. 2022;39(12):1168–72.

[28] Wang L, Hao S, Mao B, Liu X. Effect of nutrition guidance during pregnancy on delivery modes and pregnancy out-come. Maternal Child Health Care China. 2019;34(06):1234–6.

[29] Lazar VMA, Rahman S, Chowdhury NH, Hasan T, Akter S, Islam MS, Ahmed S, Baqui AH, Khanam R. Folate deficiency in pregnancy and the risk of preterm birth: A case control study. J Glob Health. 2024;14:04120. 10.7189/jogh.14.04120 PMC11239188 38991209

[30] Mao B, Shao Y, Li J, Liu X. The joint associations of folic acid supplementation and dietary folate intake with preterm birth. Chin Prev Med. 2018;19(07):491–7.

[31] De-Regil LM, Peña-Rosas JP, Fernández-Gaxiola AC, Rayco-Solon P. Effects and safety of periconceptional oral folate supplementation for preventing birth defects. Cochrane Database Syst Rev. 2015;2015(12):Cd007950. 10.1002/14651858.CD007950.pub3 PMC8783750 26662928

[32] Martínez-Frías ML. Folic acid: a public-health challenge. Lancet. 2006;367(9528):2057. 10.1016/S0140-6736(06)68917-4 16798382

[33] Wu H, Zhu P, Geng X, Liu Z, Cui L, Gao Z, Jiang B, Yang L. Genetic polymorphism of MTHFR C677T with preterm birth and low birth weight susceptibility: a meta-analysis. Arch Gynecol Obstet. 2017;295(5):1105–18. 10.1007/s00404-017-4322-z 28283826

[34] Vafapour M, Talebi H, Danaei M, Yeganegi M, Azizi S, Dastgheib SA, Bahrami R, Pourkazemi M, Jayervand F, Shahbazi A, et al. Global and population-specific association of MTHFR polymorphisms with preterm birth risk: a consolidated analysis of 44 studies. BMC Pregnancy Childbirth. 2025;25(1):230. 10.1186/s12884-025-07378-6 PMC11871749 40025425

[35] Turgal M, Gumruk F, Karaagaoglu E, Beksac MS. Methylenetetrahydrofolate Reductase Polymorphisms and Pregnancy Outcome. Geburtshilfe Frauenheilkd. 2018;78(9):871–8. 10.1055/a-0664-8237 PMC6138472 30258247

[36] Jose S. Maternal methylenetetrahydrofolate reductase (MTHFR) A1298C polymorphism: implications in preventing recurrent pregnancy loss. J Prev Med Hyg. 2024;65(1):E1–3. 10.15167/2421-4248/jpmh2024.65.1.3079 PMC11066823 38706759

[37] Memon SI, Acharya NS. The Association Between Serum Homocysteine Levels and Placenta-Mediated Complications: A Narrative Review. Cureus. 2022;14(11):e31305. 10.7759/cureus.31305 PMC9733802 36514664

[38] Rosario FJ, Nathanielsz PW, Powell TL, Jansson T. Maternal folate deficiency causes inhibition of mTOR signaling, down-regulation of placental amino acid transporters and fetal growth restriction in mice. Sci Rep. 2017;7(1):3982. 10.1038/s41598-017-03888-2 PMC5479823 28638048

[39] Froese DS, Fowler B, Baumgartner MR. Vitamin B(12), folate, and the methionine remethylation cycle-biochemistry, pathways, and regulation. J Inherit Metab Dis. 2019;42(4):673–85. 10.1002/jimd.12009 30693532

[40] Wilson A, Chiu YM, Hsu HL, Wright RO, Wright RJ, Coull BA. Potential for Bias When Estimating Critical Windows for Air Pollution in Children’s Health. Am J Epidemiol. 2017;186(11):1281–9. 10.1093/aje/kwx184 PMC5860147 29206986

